# Retinal and Choroidal Alterations in Thyroid-Associated Ophthalmopathy: A Systematic Review

**DOI:** 10.3390/life15020293

**Published:** 2025-02-13

**Authors:** Alexandra Magdalena Ioana, Diana Andrei, Daniela Iacob, Sorin Lucian Bolintineanu

**Affiliations:** 1 Doctoral School, “Victor Babes” University of Medicine and Pharmacy Timisoara, Eftimie Murgu Square No. 2, 300041 Timisoara, Romania; alexandra.ioana@umft.ro; 2 Department of Anatomy and Embriology, “Victor Babes” University of Medicine and Pharmacy Timisoara, 300041 Timisoara, Romania; s.bolintineanu@umft.ro; 3 Department of Balneology, Medical Rehabilitation and Rheumatology, “Victor Babes” University of Medicine and Pharmacy Timisoara, 300041 Timisoara, Romania; 4 Department of Neonatology, “Victor Babes” University of Medicine and Pharmacy Timisoara, Eftimie Murgu Square No. 2, 300041 Timisoara, Romania; iacob.daniela@umft.ro

**Keywords:** thyroid-associated ophthalmopathy, thyroid eye disease, Graves’ ophthalmopathy, Graves’ disease, optical coherence tomography, retina, choroid, retinal nerve fiber layer, subfoveal choroidal thickness

## Abstract

Thyroid-associated ophthalmopathy (TAO), or Graves’ orbitopathy (GO), is a complex autoimmune disorder affecting orbital tissues, often leading to vision-threatening complications such as dysthyroid optic neuropathy (DON). In this systematic review, conducted following PRISMA guidelines, 22 studies were evaluated to investigate the role of optical coherence tomography (OCT) in assessing retinal and choroidal changes in TAO. Parameters such as the retinal nerve fiber layer (RNFL), ganglion cell complex (GCC), ganglion cell layer (GCL), and choroidal thickness were analyzed. RNFL changes varied by disease severity, with significant thinning in DON due to nerve fiber loss and thickening in early DON due to optic disk edema. Subfoveal choroidal thickness (SFCT) was consistently higher in active TAO, correlating positively with the clinical activity score (CAS) and proptosis, suggesting its role as a marker of disease activity. Subgroup analysis revealed that spectral-domain OCT (SD-OCT) was the most sensitive for detecting retinal changes. The findings highlight the effectiveness of OCT in detecting minor retinal and choroidal alterations in TAO. However, the variability of study designs, as well as the lack of longitudinal data, limits the ability to draw broad conclusions. Further standardized, long-term investigations are required to properly understand OCT’s diagnostic and prognostic value in TAO.

## 1. Introduction

Thyroid-associated ophthalmopathy (TAO), or Graves’ orbitopathy (GO), is a complex autoimmune disorder that primarily affects the orbital and periorbital tissues. About 40% of individuals with Graves’ disease present with GO, making it the most prevalent extra-thyroidal manifestation of Graves’ disease [1]. TAO has a profound impact on patients’ quality of life due to its cosmetic and functional consequences, including diplopia, proptosis, and vision-threatening complications such as compressive optic neuropathy [2]. The expected prevalence of TAO nowadays is lower than earlier estimates [3] and ranges from 3.3 to 8.0/100,000/year for women and 0.9 to 1.6/100,000/year for males, with a higher incidence in middle-aged individuals [4]. Despite its relatively low prevalence, TAO imposes a significant healthcare burden. While it is more common in women, the gender disparity decreases in severe GO [5]. About half of the patients had clinically significant TAO in a study of over 2000 Graves’ patients; however, patients with moderate-to-severe TAO were more likely to be elderly and male (30% vs. 21%) [6].

The pathophysiological mechanisms of TAO are varied, involving the autoimmune activation of orbital fibroblasts by autoantibodies against the thyroid-stimulating hormone receptor (TSHR) and insulin-like growth factor 1 receptor (IGF-1R) [7,8]. This leads to inflammation, adipogenesis, and glycosaminoglycan deposition within orbital tissues, resulting in orbital expansion and increased pressure [7]. The extraocular muscles and orbital adipose tissue are the most severely afflicted areas. While the orbital tissues are the primary targets, TAO’s effects extend beyond the orbit, potentially impacting the optic nerve and retinal structures [9]. Mechanical compression of the optic nerve at the apex and inflammatory mediators can disrupt retinal microstructures, such as the retinal nerve fiber layer (RNFL), ganglion cell complex (GCC), and ganglion cell layer (GCL) [9,10]. Though less studied than the disease’s orbital signs, neuro-ophthalmic alterations may be crucial to the visual impairment linked to TAO.

TAO typically manifests within 18 months of the onset of thyroid disease, although it may manifest years afterward. The course of TAO is consistent with a predictable trajectory, during which active disease persists for 6–18 months. After that, the disease becomes less severe before plateauing during the quiescent period [11]. The clinical activity score (CAS), first used in 1989, remains the standard tool for assessing disease activity [12]. It was developed as a clinical classification to help distinguish between the active and quiescent stages of the disease [13]. While the majority of individuals have a benign course, approximately 5% acquire dysthyroid optic neuropathy (DON), a significant condition that may result in retinal ganglion cell death and permanent vision impairment [14]. Imaging modalities, such as optical coherence tomography (OCT), and the newly developed OCTA, have emerged as valuable tools for providing objective, quantifiable insights into TAO-related changes. OCT provides a view into the subtle neuro-ophthalmic changes linked to TAO by allowing the precise observation and quantification of retinal structures [15].

According to latest research studies, biomarkers obtained via OCT, such as RNFL thinning, GCC loss, and GCL changes, may be used to detect involvement of the optic nerve before clinically apparent optic neuropathy manifests [16,17]. These biomarkers could be helpful in disease severity stratification and progression prediction in addition to their diagnostic capacities. Furthermore, since retinal and optic nerve abnormalities can happen even in patients with low CAS, OCT results may support clinical evaluations [15].

Despite these developments, it is still unclear how OCT results correspond to the clinical characteristics of TAO. The necessity for a thorough synthesis of the available information is demonstrated by the variation in OCT results between studies, which is impacted by variables such as disease stage and severity or patient demographics.

Additionally, the connection between orbital inflammation, mechanical effects, and neurodegeneration in TAO raises important questions about the pathophysiological mechanisms leading to these changes.

This systematic review aims to synthesize current evidence on the role of OCT in assessing TAO, with a focus on changes in the choroidal thickness, RNFL, GCC, and GCL thickness. It also explores the clinical implications of these findings, examining their relationship with disease activity and severity. By integrating data from diverse studies, this review seeks to provide a comprehensive overview of how OCT can develop the understanding of TAO and its impact on ocular structures.

## 2. Materials and Methods

### 2.1. Study Design and Protocol Registration

This systematic review was conducted in accordance with the Preferred Reporting Items for Systematic Reviews and Meta-Analyses (PRISMA) guidelines [18]. The protocol for the review was registered in the PROSPERO database (CRD42025638423). The primary aim of the review was to evaluate changes in the retinal nerve fiber layer (RNFL), ganglion cell complex (GCC) or ganglion cell layer (GCL) thickness, and choroidal thickness in patients with TAO using OCT.

### 2.2. Search Strategy

A comprehensive literature search was performed across major databases: PubMed, Google Scholar and Scopus in the last ten years (last search date: 2 January 2025). The search strategy combined keywords and Medical Subject Headings (MeSH) terms, such as “Thyroid associated ophthalmopathy”, “Thyroid eye disease”, “Graves’ orbitopathy”, “Optical coherence tomography”, “Retinal nerve fiber layer”, “Ganglion cell complex”, “Ganglion cell layer”, “Choroidal thickness”, and “Retinal parameters”. Boolean operators were used to combine the terms (“AND”, “OR”), and manual searches of reference lists from relevant articles and reviews were also conducted to identify additional studies.

### 2.3. Inclusion and Exclusion Criteria

This study included observational designs, such as cross-sectional, case–control, and cohort studies, that assessed retinal and choroidal thickness in TAO patients versus healthy controls. All stages of TAO, from mild to severe, included DON. Studies were eligible if they reported at least one measurement of the RNFL, GCC/GCL, or choroidal thickness. Only studies providing quantitative data on these OCT parameters were included. English-language studies were selected to standardize the review process and avoid language bias. Additional inclusion and exclusion criteria are listed in Table 1. This study was also accomplished using the PICO (Population, Intervention, Comparison, Outcome) framework [19] to guide the selection and evaluation of studies (Table 2).

Two independent reviewers screened titles and abstracts against the eligibility criteria. Full-text articles of potentially relevant studies were then assessed for inclusion. Disagreements between reviewers were resolved by discussion or consultation with a third reviewer to ensure objectivity.

**Figure 1:**
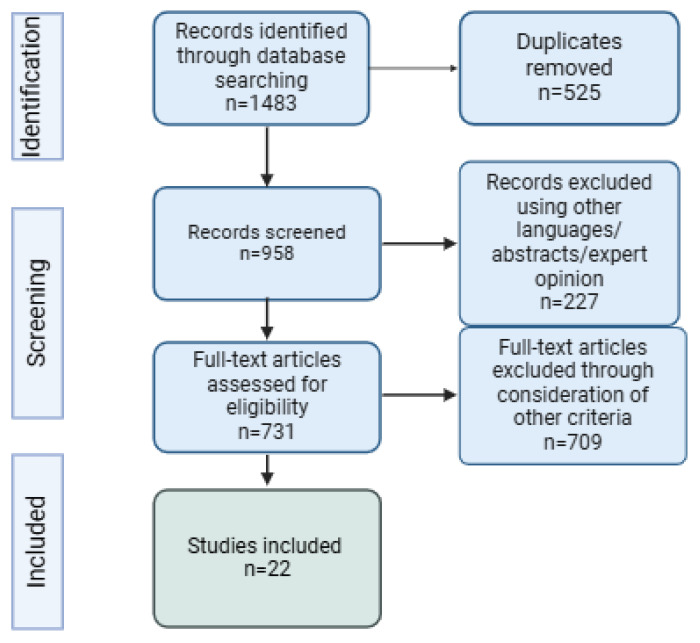
PRISMA flowchart.

Figure 1 shows the PRISMA flowchart, which details the systematic process of study selection for this review.

### 2.4. Data Extraction and Management

Two reviewers extracted data using a pre-designed collection form. Extracted information included study characteristics (author, year, country, and study design), patient demographics (sample size, age, gender, TAO activity or severity), OCT parameters (RNFL, GCC, and GCL thickness, choroidal thickness), comparator group details (healthy controls or TAO subgroups), and outcomes (mean thickness values, correlations with clinical activity). Methodological quality indicators, including participant selection and assessment protocols, were also recorded. If studies reported data in different units or scales, all measurements were converted to the same unit. Variations in terminology across studies were addressed by thoroughly reviewing each study’s definitions and methodologies to ensure consistency, and a unifying term was applied to synthesize the data. Outliers in the data were reviewed and analyzed separately to see if they affected the overall results.

### 2.5. Risk of Bias Assessment

The methodological quality of included studies was assessed independently by two reviewers. Cross-sectional, case–control, and cohort studies were evaluated using the original or adapted (in the case of cross-sectional studies) Newcastle-Ottawa Scale (NOS) [20]. Each study was rated as low, moderate, or high risk of bias, with the latter being excluded from this systematic review.

### 2.6. Data Synthesis

A narrative synthesis was conducted to summarize findings across studies. The authors did not perform a meta-analysis due to the lack of standardization among the study groups and the variability in reported parameters. The included studies assessed different outcomes and used inconsistent group definitions, making it unlikely for a meta-analysis to achieve statistical significance or provide meaningful pooled results. The primary outcomes of interest were differences in RNFL, GCC/GCL thickness and choroidal thickness between TAO patients and controls or across TAO subgroups. Secondary outcomes included correlations between retinal layer changes and clinical activity scores/clinical changes. A subgroup analysis was conducted, which involved examining the impact of the OCT type on reported outcome retinal parameters (RNFLs). To present the results of studies, tables that systematically listed all available data were used.

To address any potential bias from unpublished or selectively reported data, the results were compared with findings from systematic reviews or meta-analyses on related topics to identify any significant discrepancies.

### 2.7. Ethical Considerations

Since this systematic review involves the analysis of published data rather than primary data collection, ethical approval was not required. All the studies included in this review were obtained from publicly available, peer-reviewed journals, and their original authors had already adhered to the ethical guidelines and protocols established by the respective ethics committees and institutional review boards at the time of their studies.

## 3. Results

This review analyzed 22 studies that used OCT in order to evaluate structural alterations in the retina and choroid in TAO studies included eight cross-sectional investigations, eight prospective studies, three case–control studies, and three retrospective studies (Table 3).

The studies were predominantly from Asia, with 11 from China, 4 from Turkey, 2 from India, and 1 each from South Korea, Hong Kong, and Iran. Europe contributed two studies from Italy, while the Middle East and Africa were represented by one study each from Israel and Egypt.

Several OCT modalities were used in the investigations: spectral-domain OCT (SD-OCT) was used in thirteen studies; swept-source OCT (SS-OCT) was used in four studies; high-definition OCT (HD-OCT) was used in three studies, and time-domain OCT (TD-OCT) was used in two studies. Two of the studies also involved OCTA as an additional method of investigation.

The studies concentrated on retinal and choroidal alterations, thirteen of them studying only retinal parameters, eight studies investigated choroidal parameters, and one of them studied both. Parameters were examined across various stages of TAO, with studies reporting the following stages or severity of disease: mild/moderate/severe, mild/moderate to severe/DON, active/inactive, and active/stable.

### 3.1. Association Between TAO and Choroidal Parameters

A total of nine studies investigated choroidal thickness changes in TAO compared to controls (Table 4), examining 294 TAO patients and 331 controls. The studies focused on subfoveal choroidal thickness (SFCT) as a key indicator of choroidal alterations in TED (five studies), with some also reporting mean choroidal thickness (MCT) (three studies) and one study reporting both of the parameters that were assessed in this review. Subfoveal choroidal thickness was generally higher in TAO patients compared to controls, with the thickening more pronounced in active TAO. Caliskan et al. reported significantly increased SFCT in active TAO patients (395.84 ± 9.68 µm) compared to controls (314.22 ± 5.74 µm) [22], while Yu et al. observed SFCT values of 313.47 ± 100.32 µm in TAO patients compared to 256.33 ± 50.18 µm in controls [40]. Similarly, Noce et al. highlighted variability in SFCT across mild, moderate, and especially severe TAO [34].

Some studies also assessed MCT, along with different quadrants of the choroid. Yining Dai et al. found significantly lower MCT in controls (236.86 ± 45.02 µm) compared to TAO patients (276.25 ± 58.75 µm) [24]. However, not all studies observed significant differences. Casini et al. reported no notable difference in SFCT between TAO patients (288 ± 88 µm) and controls (287 ± 58 µm). They were the only research group to report both MCT and SFCT and did not report important differences between groups [23].

Most studies demonstrated statistically significant differences in SFCT or MCT between TAO patients and controls, with *p*-values often below 0.05. Active TAO patients consistently showed greater SFCT compared to inactive TED or controls, indicating a potential link between choroidal thickening and inflammatory activity.

A regional analysis of the studies included revealed some minor variations in choroidal thickness findings. Studies conducted in Asia consistently reported significantly increased SFCT in active TAO patients. In contrast, one study from Europe did not reveal important differences among different groups.

### 3.2. Association Between TAO and Retinal Parameters

#### 3.2.1. Retinal Nerve Fiber Layer

A total of 10 studies explored the relationship between retinal nerve fiber layer thickness and TAO, examining 443 patients with TED and 391 healthy controls (Table 5). Several of them also reported variations across different disease stages. These studies compared RNFL thickness in TAO patients with healthy controls and assessed its association with disease severity.

Overall, RNFL thickness in TED patients often showed no significant differences compared to controls in mild and moderate stages but tended to increase in DON due to optic disk edema in a few of the cases. One study showed an important decrease in patients with DON as compared to all the other study groups, most probably patients with advanced cases due to nerve fiber loss [28]. Significant RNFL changes were more frequently observed when comparing DON to control groups or moderate TAO.

Several studies demonstrated significant RNFL differences. Abdolizadeh et al. reported a significant difference in RNFL thickness between DON patients and both mild TAO and controls [21]. Luo et al. found significant RNFL thinning in DON patients compared to controls (*p* = 0.003) [31], while Jian et al. identified substantial thinning in DON (63.47 ± 15.81 µm) compared to controls and non-DON groups (*p* < 0.001) [28]. Mugdha et al. also showed significant RNFL thinning (*p* = 0.0001) in TAO patients compared to controls, particularly in advanced cases. They also studied the RNFL in different age groups and showed that it decreased significantly with age, particularly between the 10–19 and 20–30 age groups (*p* = 0.005), with a progressive decline observed in older age groups [33]. Guo et al. showed that the mean RNFL thickness was thinnest in the moderate-to-severe TAO group (97.8 ± 9.2 µm) and thickest in the DON group (110.6 ± 34.2 µm), with a significant difference between these groups (*p* = 0.036). However, they reported no significant difference between any TAO stage and healthy patients [27]. Some studies, such as those by Park et al. and Wu et al., found no significant RNFL differences between TAO patients and controls in the early stages of the disease [36,38]. Xu et al. reported RNFL thickening in active TAO (114.93 ± 7.58 µm) compared to stable TAO (103.74 ± 8.40 µm) and controls [39].

A subgroup analysis was conducted to assess the impact of the type of OCT used on RNFL thickness measurements in TAO and control groups. Studies were categorized based on whether they used spectral-domain OCT (SD-OCT), high-definition OCT (HD-OCT), or time-domain OCT (TD-OCT). In the first type of OCT, for SD-OCT, six studies were included. In the control group, RNFL thickness ranged from 100.3 ± 6.3 µm [27] to 118.01 ± 13.07 µm [28], with a pooled estimate of 104.73 ± 11.61 µm. In the TAO groups, RNFL thickness varied significantly based on disease severity. These studies consistently demonstrated significant RNFL thinning in moderate-to-severe TED and DON cases. For HD-OCT investigation, three studies were included. In the control group, RNFL thickness ranged from 96 ± 8 µm [36] to 101.28 ± 6.64 µm [33], with a pooled estimate of 99.86 ± 5.79 µm. In the TAO group, RNFL thickness showed slight reductions. For TD-OCT, two studies were included. In the control group, there was a pooled estimate of 99.25 ± 16.95 µm. In the TAO group, RNFL thickness showed minimal reductions. Overall, SD-OCT was the most sensitive method for detecting RNFL thinning, while HD-OCT and TD-OCT were less sensitive.

Seven studies examined RNFL thickness across quadrants, showing variations among TAO patients. Guo et al. reported no statistically significant differences across quadrants among TAO stages, although the inferior RNFL thickness was 126.7 ± 15.9 µm in the moderate-to-severe group and 147.2 ± 50.3 µm in the DON group (*p* = 0.193). In the nasal quadrant, RNFL thickness increased from 68.7 ± 9.4 µm in the moderate-to-severe group to 80.1 ± 28.6 µm in the DON group (*p* = 0.087) [27]. Mugdha et al. observed significant RNFL thinning in all quadrants compared to controls (*p* < 0.01). However, at follow-up, no significant changes were noted (*p* > 0.05) except for the inferior quadrant, which showed significant thinning (*p* = 0.02) [33]. Luo et al. found reduced temporal and nasal RNFL thicknesses in TED patients compared to controls (*p* = 0.041, *p* = 0.012). In DON patients, temporal-inferior and nasal-superior quadrants demonstrated significant thickening [31]. Jian et al. reported significant differences in the superior, inferior, and nasal quadrants between DON patients and other groups (*p* < 0.001) [28]. Wu et al. observed significant thinning of RNFL in the superior, inferior, and nasal quadrants in DON patients compared to controls and non-DON groups (*p* < 0.001), with additional thinning in the temporal quadrant (*p* = 0.001) [38]. Park et al. found significant differences in temporal peripapillary RNFL thickness among eyes with acute DON, chronic DON, and controls (*p* = 0.014) [36]. In contrast, Sayin et al. reported no significant differences in RNFL thickness across quadrants between TED patients and controls [37].

#### 3.2.2. Central Retinal Thickness

Five studies investigated central retinal thickness (CRT) in TAO (Table 5). Additionally, only one study reported both parameters discussed, the RNFL and CRT. Casini et al. reported significantly reduced CRT in the TED group (275 ± 19 µm) compared to healthy controls (285 ± 20 µm) (*p* = 0.017) [23]. Sayin et al. observed significant CRT thinning in the patient group compared to controls (*p* < 0.05), with reductions noted in both the right and left eyes and across all quadrants when both eyes were assessed together (*p* < 0.05) [37]. Xu et al. found no statistically significant differences in CRT between active TAO (241.07 ± 35.83 μm), stable TED (242.65 ± 13.44 μm), and healthy controls (251.64 ± 14.44 μm) (*p* = 0.160) [39].

A notable regional trend was found regarding RNFL and CRT measurements. Among the included studies, all four that reported no statistically significant difference in RNFL or CRT between TAO patients and controls were conducted in Asian populations. However, out of the 13 studies that investigated RNFL and CRT, only two were conducted outside of Asia.

**Table 5:** Studies investigating RNFL and/or CRT in thyroid-associated ophthalmopathy.

Study	RNFL Control Group	RNFL TED Group	CRT Control Group	CRT TED Group	*p*-Value	Observations
Abdolizadeh et al. [21]	101.97 ± 8.93	98.99 ± 14.96	-	-	<0.05	Analyzed mild/moderate/DON disease stage Globally significant difference between DON and both mild TAO and control
Casini et al. [23]	-	-	287 ± 20	275 ± 19	0.008	Also investigated Graves’ disease without TAO
Elsamkary et al. [26]	-	-	257.35 ± 16.97	247.53 ± 9.63	0.002	No significant difference in mean outer macular thickness
Guo et al. [27]	100.3 ± 6.3	103.2 ± 6.6 (mild)/97.8 ± 9.2 (moderate to severe)/110.6 ± 34.2 (DON)	-	-	>0.05 in all cases (TED stages vs. control)	Significant difference between moderate-to-severe group and DON groups
Jian et al. [28]	118.01 ± 13.07	63.47 ± 15.81 (DON)/118.68 ± 11.08 (inactive non-DON)/122.79 ± 15.33 (active non-DON)	-	-	<0.001	Total and regional RNFL thickness were similar among the non-DON, active non-DON, and healthy control groups
Luo et al. [31]	103.15 ± 9.29	105.17 ± 13.21 (mild)/101.05 ± 10.28 (moderate)/111.38 ± 7.74 (DON)	-	-	0.003	Significant between DON and control. No significant differences between the other groups
Meirovitch et al. [32]	96.25 ± 9.42	100.06 ± 33.3	-	-	<0.05	No significant increase n the temporal region
Mugdha et al. [33]	101.28 ± 6.64	92.06 ± 12.44	-	-	0.0001	-
Park et al. [36]	96 ± 8	101 ± 11 (acute DON)/96 ± 11 (chronic)	-	-	0.270	No significant increase in RNFL between acute DON patients and controls
Sayin et al. [37]	102.8 ± 29.7 (RE)/100.7 ± 31.1 (LE)	99.9 ± 29.3 (RE)/96.7 ± 29.4 (LE)	245.6 ± 27.9 (RE)/247.6 ± 35.3 (LE)	238.6 ± 29.7 (RE)/240.0 ± 30.0 (LE)	>0.05 (RNFL)/0.01 (CRT)	When both eyes were analyzed, only the inferior RNFL values showed a significant difference, being thinner in the patient group compared to controls
Wu et al. [38]	-	-	213.3 ± 51.3	214.7 ± 14.9 (non-DON)/215.8 ± 36.9 (DON)	>0.7 in all cases	-
Xu et al. [39]	108.03 ± 9.15	114.93 ± 7.58 (active)/103.74 ± 8.40 (stable)	251.64 ± 14.44	241.07 ± 35.83 (active)/242.65 ± 13.44 (stable)	<0.001 (RNFL) 0.160 (CRT)	For RNFL: active vs. stable <0.001; active vs. control 0.005; stable vs. control 0.175
Zhang et al. [41]	104.91 ± 7.99	101.36 ± 8.64 (non-DON)/98.30 ± 10.77 (DON)	-	-	0.04	-

Abbreviations: CRT—central retinal thickness; DON—dysthyroid optic neuropathy; LE—left eye; RE—right eye; and RNFL—retinal nerve fiber layer.

#### 3.2.3. Ganglion Cell Layer/Ganglion Cell Complex

Seven studies investigated changes in the ganglion cell complex (GCC) or ganglion cell layer (GCL) in TAO (Table 6). Luo et al. reported no significant differences in macular GCC thickness between various severity groups and the control group (*p* > 0.05) [31]. Zhang et al. found significant differences in GCC thickness among groups, with DON eyes exhibiting thinner mean GCC thickness compared to non-DON eyes (*p* = 0.008, post hoc *p* = 0.026) [41]. Guo et al. observed the lowest mean GCL/IPL thickness in the DON group compared to mild, moderate-to-severe, and control groups (all *p* < 0.001), with significant reductions in the moderate-to-severe group compared to controls (*p* < 0.001) [27]. Casini et al. demonstrated significantly reduced central GCL thickness in TAO patients (14.87 ± 3.0 µm) compared to controls (17.92 ± 5.02 µm, *p* = 0.001), and this reduction was also significant compared to patients without TAO (*p* = 0.001) [23].

Kurt et al.’s study was not included in the table as it focused on the thickness of different quadrants of the GCL. Their findings showed no significant differences in the mean GCL thickness measurements in between the study and control groups [29].

### 3.3. Connection Between OCT Changes and Clinical Aspects

Seven studies have explored correlations between clinical aspects of TAO/Graves’ disease and retinal or choroidal parameters

Proptosis, measured through exophthalmometry, has been believed to have a variable impact on retinal and choroidal thickness. Elsamkary et al. identified a significant negative correlation between proptosis and central foveal thickness, with greater proptosis associated with thinner central foveal thickness in TAO (r = −0.59, *p* < 0.0001) [26]. Lai et al. further confirmed this association, reporting consistent negative correlations between subfoveal choroidal thickness and exophthalmometry in both univariate (β = −5.56, *p* = 0.038) and multivariate analyses (β = −5.04, *p* = 0.043), highlighting the link between proptosis severity and choroidal thinning [30].

Choroidal thickness has also been examined in relation to the CAS. Caliskan and Dave found significant positive correlations between subfoveal choroidal thickness and CAS in univariate analyses. However, in multivariate models, these associations were influenced by collinearity with other factors [22,25]. In the univariate analysis of Caliskan et al., thicker subfoveal choroidal thickness was significantly associated with higher clinical activity scores (*p* < 0.001) and greater proptosis (*p* < 0.001). However, in the multivariate analysis, the CAS was excluded due to high collinearity (variance inflation factor [VIF]: 8.04), and proptosis was removed as it was no longer significantly associated with subfoveal CT (*p* = 0.466) [22]. In a study conducted by Dave et al. [25], the CAS demonstrated a significant positive association with choroidal thickness in bivariate analysis (coefficient = 11.92 ± 5.17, *p* = 0.03). However, this association became borderline non-significant in multivariate analysis (coefficient = 11.89 ± 6.76, *p* = 0.08 [25]. Del Noce et al. reported a strong positive correlation between choroidal thickness and the EUGOGO clinical score (Pearson r = 0.84, *p* < 0.000001) [34]. Mugdha’s findings indicated no meaningful correlation between the CAS and RNFL thickness at any time point (r = −0.13 at the first visit and r = −0.02 at the second visit). Similarly, changes in the CAS and RNFL over time showed no meaningful correlation (r = +0.14), indicating no direct relationship between disease activity and RNFL thickness [33].

In a study conducted by Guo et al., there were significant differences in the mean CAS between the three groups (*p*  <  0.001). The mean proptosis was substantially higher in DON and moderate-to-severe groups compared to the mild group (*p*  <  0.05). The DON group had significantly worse best-corrected visual acuity (BCVA) compared to the mild and moderate-to-severe groups (*p*  <  0.001) [27].

Additionally, Park et al. described that, among acute DON patients, treatments varied; patients received orbital radiotherapy, orbital decompression, steroids with radiotherapy, and combined therapies. In chronic DON, they received steroids, radiotherapy, or underwent decompression. At the follow-up consult, 81% of DON eyes achieved 20/20 vision, with significant improvement (*p* < 0.001). Visual acuity improved by ≥3 logMAR lines in 50% of acute and 32% of chronic DON cases [36].

### 3.4. Study Bias Assessment

The risk of bias for all 22 studies in this systematic review was evaluated, with a summary of the assessment presented in Table S1. For the studies included, the original and adapted Newcastle-Ottawa Scale (NOS) was applied. In the analysis, many cross-sectional studies showed moderate comparability, indicating partial control of confounders but leaving room for residual bias. This might limit the ability to attribute outcomes solely to the exposure of interest, affecting the overall reliability. Selection bias was present in studies with small sample sizes or inadequate randomization methods (five studies), while performance bias was identified in studies where the blinding of assessors or participants was not explicitly reported, which may have influenced outcome measurements (three studies). Additionally, attrition bias was noted in studies with incomplete follow-up data or high dropout rate (two studies). Despite these limitations, the overall quality of the included studies was moderate to high, supporting the reliability of the observed structural changes in TAO. Among the 22 studies, 14 were graded as low risk, 6 were graded as low/moderate risk, and the remaining 2 were graded as moderate risk of bias.

## 4. Discussion

This review analyzed 22 studies using OCT to evaluate retinal and choroidal changes in TAO and its stages. The studies, predominantly from China and Turkey, included various designs, with most focusing on the RNFL and choroidal thickness. Five studies reported CRT values in TAO patients, ten examined the RNFL, showing stage-dependent differences, particularly in DON, while six explored GCL and GCC alterations. Nine studies highlighted subfoveal choroidal thickness (SFCT) in TAO patients, and six studies proposed and studied the CAS and proptosis as influencing factors. There is an extremely low number of systematic reviews that describe retinal/choroidal alterations using OCT in TAO patients. Unlike prior reviews, this systematic analysis specifically focuses on the role of OCT in assessing both retinal and choroidal changes. A comprehensive evaluation of the RNFL, GCC/GCL, SFCT and other relevant parameters was conducted, with particular emphasis on their relationship with disease activity and severity.

The research of choroidal and retinal modifications in TAO has improved greatly since the introduction of OCT, which allows for the high-resolution, non-invasive imaging of the retinal and choroidal layers [15]. OCT allows for a thorough investigation of structural changes, which provides understanding of vascular and inflammatory processes related with TAO [15,39]. However, variations in imaging procedures, patient demographics, and disease severity between studies have resulted in heterogeneous findings. Longitudinal studies using standardized approaches would be required for understanding the occurrence of these changes in TAO and their clinical consequences.

RNFL alterations have been extensively studied in TAO as a marker of optic nerve involvement and disease severity [43]. The findings across studies reveal variability in RNFL thickness depending on the stage and severity of TAO [41]. While many studies report no significant RNFL changes in mild or moderate TAO compared to controls, advanced cases, particularly those involving dysthyroid optic neuropathy (DON), often show distinct RNFL alterations [28]. In DON, RNFL thickening may occur due to optic disk edema caused by increased intraorbital pressure or inflammation. It is also plausible that an interaction of compressive mechanical and inflammatory factors may obscure RNFL alterations [32]. Chronic or advanced stages of TAO, prolonged compression of the optic nerve due to enlarged extraocular muscles, increased retrobulbar pressure, or apical crowding can lead to ischemia and axonal loss [44,45], as seen in a study conducted by Jian et al. [26]. This results in RNFL thinning, particularly in the inferior and nasal quadrants, which are more vulnerable to mechanical compression. The degree of RNFL thinning is often correlated with the severity of visual impairment, making it a valuable parameter for assessing disease progression and predicting visual outcomes [46]. One systematic review discussed retinal layer thickness. They found no differential reduction in RNFL thickness in the superior areas. They reported a similar number of studies revealing thinning of the superior (six studies), inferior (seven studies), nasal (five studies), and temporal (six studies) RNFLs in TAO patients [43]. Chien et al. also discussed choroidal parameters in a systematic review. Similarly to our results, they reported an increased choroidal thickness in TED patients compared to normal controls, particularly in the subfoveal region. Furthermore, some of the studies included in the review found that patients with active TED had an increased thickness than those with inactive TED; however, the results were mixed. Finally, numerous studies found links between increased choroidal thickness and worsening clinical measures of disease activity, such as the CAS [47]. Another systematic review and meta-analyses on SFCT reported statistically significant differences in terms of the SFCT parameter between the study and control groups and also between active and inactive forms of TED [48]. Goel et al. performed a systematic review of orbital and ocular perfusion in TED. The majority of studies reporting SFCT indicated choroidal thickening in active TED. The authors hypothesized that choroidal thinning, which has been shown in just a few studies, could be caused by homeostatic changes or paradoxical compression effects [49]. The same important aspect observed in this systematic review was reported in other systematic reviews, such as the ethnicity of participants (particularly Asian populations), which may be associated with differences in orbital morphology.

RNFL changes in TAO might also influenced by other factors such as age, gender, intraocular pressure and proptosis [28,50]. However, several studies found no significant differences in RNFL thickness between the study groups, despite elevated intraocular pressure in one of the groups [51]. Other investigations found RNFL thinning in TAO patients without clinical optic nerve impairment, notably in the inferior quadrant [35,52]. This raises the notion that RNFL degradation occurs independently of IOP and that structural RNFL abnormalities may exist even in clinically normal conditions. A potential correlation between the CAS and RNFL thickness in TAO has been explored in several studies. However, the findings regarding this correlation remain inconsistent. In a study conducted by Sarnat-Kucharczyk et al., RNFL thickness showed a slight decrease after treatment, with a reduction of ≥2 μm observed in three patients. This change occurred despite improvements in the CAS, which showed a median reduction of 4 points over 48 weeks [53]. Some studies suggest no significant relationship between the CAS and RNFL thickness, indicating that inflammatory activity measured by the CAS may not directly influence RNFL changes. Mugdha et al. found no association between thyroid disease clinical activity and RNFL status. At the six-month follow-up, the CAS improved, but RNFL damage persisted, with further thinning in the inferior quadrant, most probably due to its greater baseline thickness [33].

In a study conducted by Guo et al. [27], there was a substantial relationship between visual functions and GCL/IPL thickness rather than RNFL thickness. Previous research has found that a better maintained GCC and/or RNFL thickness indicates better vision rehabilitation. Patients with early DON might have great visual recovery following treatments, even if the thinning RNFL and GCC showed no substantial improvement [54,55]. The thinning tendency of GCL/IPL may be a significant indication for closer vision monitoring and sooner decompression surgery in TAO patients [27].

Increased intraorbital pressure induced by the enlargement of extraocular muscles and orbital fat can cause choroidal thickness changes, resulting in vascular congestion and episcleral venous stasis. In addition, inflammation has an important role, with inflammatory mediators producing exudation, vascular leakage, and abnormal ocular blood flow [56,57,58]. These factors collectively contribute to changes in CT in TED. The alterations in choroidal thickness in patients with TED were initially reported by Caliskan et al. [22]. They found that, in patients with active TED, the mean subfoveal choroidal thickness rises. They suggested that a relevant metric for tracking disease activity would be subfoveal choroidal thickness [22]. The findings of other studies have reinforced this presumption [42]. In contrast with a study conducted by Caliskan et al., where the thickness changes were greater in the temporal quadrant, Dave et al. showed greater thickness in the nasal quadrant. Even in the absence of obvious clinical symptoms, their investigation found that both the inner nasal choroidal thickness and subfoveal central choroidal thickness were reliable indicators of activity [25]. Lai et al. found that, in TAO patients, a thinner subfoveal choroidal thickness was linked to increased age and axial length, greater proptosis, and worse visual acuity [30]. According to an ROC curve analysis, SFCT outperformed retinal, RNFL, GCL, GCL+, and GCL++ thicknesses in terms of disease activity assessment [45].

The limitations of this study include the heterogeneity of the included studies, which varied in design, population size and characteristics, imaging modalities, and outcome measures. The increased heterogeneity parameters also prevented the performance of a meta-analysis, which could have provided a better summary of the findings. Additionally, geographic and demographic bias is another limitation, as the majority of studies originated from Asia, particularly China, with underrepresentation from other regions, which may affect the generalizability of the findings. Furthermore, most studies were cross-sectional, providing only a brief idea of structural changes, and the lack of longitudinal data limits insights into the progression of retinal and choroidal alterations over time. Our focus on specific OCT parameters may have overlooked other potentially relevant structural or functional aspects of disease progression. Lastly, while a comprehensive search strategy was used, there remains the possibility of missing relevant studies, especially non-English studies, which may have introduced selection bias.

Because the majority of the studies in this systematic review were cross-sectional, data from individuals with TED were collected at various stages of their disease progression, which might result in thickness heterogeneity. Therefore, future longitudinal studies should focus on standardizing OCT acquisition protocols, using uniform segmentation techniques and image analysis criteria. Additionally, prospective studies should evaluate the temporal evolution of these changes in relation to disease activity, response to therapy, and clinical outcomes. To enhance clinical applicability, CAS thresholds should be incorporated to assess the utility of OCT as a disease monitoring tool. Furthermore, given the predominance of Asian-based studies in the current literature, future research should prioritize multi-ethnic studies to evaluate potential population-based differences in retinal and choroidal changes.

## 5. Conclusions

This study focuses on important retinal and choroidal structural changes in TAO. OCT can noninvasively detect changes across various stages of TAO patients and could be implemented as a high diagnostic value tool. The observed changes in RNFL thickness, CRT, GCC/GCL, and choroidal parameters indicate that they might be indicators of disease progression and severity. The results across studies reveal variability in retinal and choroidal thickness depending on the stage and severity of TAO. However, the most important changes in these parameters were observed in the DON group when compared to controls. These findings may help improve our understanding of the underlying pathophysiology of TAO and assist in the development of imaging-based diagnostic and monitoring techniques. More research is needed to better understand the clinical significance of these structural changes and their function in guiding treatment therapies.

## Figures and Tables

**Table 1:** Inclusion and exclusion criteria.

Inclusion Criteria	Exclusion Criteria
Original, observational studies	Reviews, editorials, abstracts, expert opinions, or case reports
Studies involving adult patients (≥18 years) with a clinical diagnosis of TED, irrespective of disease activity or severity	Studies involving pediatric populations or non-thyroid-related orbital diseases
Studies included both one (or more) group of patients diagnosed with TED and a healthy control group	Studies lacked a control group of healthy individuals or did not clearly distinguish between groups in their analyses
Studies reporting quantitative data on at leat one of the following: RNFL, GCC, GCL or choroidal thickness	Studies with insufficient data on OCT measurements or missing relevant outcomes
Peer-reviewed articles published in English	Articles published in other languages

**Table 2:** PICO framework.

Population	Patients diagnosed with TAO (all stages of disease severity)
Intervention	Evaluation of structural and functional changes in the retina and choroid using OCT
Comparison	Healthy control groups with no TAO or other significant ocular conditions
Outcome	Quantitative measurements of retinal and choroidal parameters

**Table 3:** Demographic and clinical parameters of included studies.

Study	Year	Country	Type	Investigantion	Control Group Patients (Eyes)	TAO Group Patients (Eyes)	Mean Age Control	Mean Age TA)	Male/Female Control	Male/Female TAO
Abdolizadeh et al. [21]	2020	Iran	Prospective	SD-OCT	20(39)	43 (79)	37.6 ± 8.35	40.79 ± 10.34	7/13	17/19
Caliskan et al. [22]	2016	Turkey	Cross-sectional	SD-OCT	37	38	38.5 ± 11.5	46.7 ± 14.3 active/40.5 ± 10.1 inactive	5/32	6/32
Casini et al. [23]	2020	Italy	Prospective	SD-OCT	40	40	36.28 ± 5.79	39.57 ± 5.60	11/29	5/35
Dai et al. [24]	2023	China	Prospective	SS-OCT	55	55	46.37 ± 11.25	46.40 ± 11.28	14/41	18/37
Dave et al. [25]	2020	India	Prospective, cross-sectional	SS-OCT	12 (24)	33 (66)	42.75 ± 4.55	42.85 ± 10.84 (inactive)/46.22 ± 12.10 (active)	8/4	22/11
Elsamkary et al. [26]	2022	Egypt	Cross-sectional	SD-OCT	40	40	46.63 ± 13.63	46.35 ± 13.32	29/11	29/11
Guo et al. [27]	2021	China	Prospective	HD-OCT	35	75	53.3 ± 11.7	46.4 ± 13.1	13/22	25/50
Jian et al. [28]	2021	China	Cross-sectional	SD-OCT	26 (52)	43 (82)	39.88 ± 12.17	43.39 ± 11.09	17/26	10/16
Kurt et al. [29]	2021	Turkey	Cross-sectional	SS-OCT	30	29	42.60 ± 12.38	40.66 ± 11.83	18/12	15/14
Lai et al. [30]	2019	Hong-Kong	Prospective	SD-OCT	26	52	45.2 ± 15.6	47.4 ± 13.2	10/16	17/35
Luo et al. [31]	2022	China	Case–control	SD-OCT	58	58	44.60 ± 11.40	43.80 ± 12.40	35/23	35/23
Meirovitch et al. [32]	2017	Israel	Case–control	TD-OCT	41 (73)	21 (42)	42.90 ± 14.65	44.19 ± 14.21	9/32	4/17
Mugdha et al. [33]	2016	India	Case–control	HD-OCT	40	40	-	-	18/22	18/22
Noce et al. [34]	2020	Italy	Cross-sectional	SD-OCT	18	18	26.50 ± 3.50	26.50 ± 4.90	4/14	7/11
Ogmen et al. [35]	2022	Turkey	Prospective	SD-OCT	18	23	35.90 ± 8.70	38.78 ± 10.80	8/15	7/11
Park et al. [36]	2016	South Korea	Cross-sectional	HD-OCT	47 (94)	30 (52)	48 ± 14	54 ± 11	26/21	15/15
Sayin et al. [37]	2016	Turkey	Cross-sectional	TD-OCT	66	36	38.30 ± 9.70	40.10 ± 12.60	11/25	23/43
Wu et al. [38]	2020	China	Prospective	SD-OCT	38 (38)	44 (74 eyes)	45.20 ± 10.8	41.90 ± 11.7 (non-DON)/47.60 ± 6.50 (DON)	19/19	23/21
Xu et al. [39]	2023	China	Cohort longitudinal retrospective	SD-OCT	39	51	44.72 ± 9.71	49.61 ± 12.42(active)/44.17 ± 10.89 (stable)	18/21	16/35
Yu et al. [40]	2018	China	Cross-sectional	SD-OCT	36	36	38.70 ± 14.5	39.80 ± 14.5	9/27	9/27
Zhang et al. [41]	2019	China	Retrospective observational	SD-OCT	19 (23 eyes)	46 (71 eyes)	46.7 ± 13.00	49.50 ± 9.00	10/9	21/25
Zhong et al. [42]	2022	China	Cross-sectional	SS-OCT	40 (67)	30 (6)	46.54 ± 18.04	45.23 ± 11.92	12/26	8/22

Abbreviations: HD OCT—high-definition optical coherence tomography; SD-OCT—spectral-domain optical coherence tomography; SS-OCT—swept-source optical coherence tomography; TAO—thyroid-associated ophthalmopathy; and TD-OCT—time-domain optical coherence tomography.

**Table 4:** Studies investigating choroidal thickness in thyroid-associated ophthalmopathy.

Study	Mean Choroidal Thickness Control Group	Mean Choroidal Thickness TAO Group	Subfoveal Choroidal Thickness Control Group	Subfoveal Choroidal Thickness TAO Group	*p*-Value	Active Disease	Inactive Disease	Observations
Caliskan et al. [22]	-	--	314.22 ± 5.74		<0.001	395.84 ± 9.68	319.76 ± 7.07	SFCT showed postivie correlation with CAS
Casini et al. [23]	269 ± 42	271 ± 72	287 ± 58	288 ± 88	0.88/0.98	-	-	Also investigated GO without TED
Dai et al. [24]	236.86 ± 45.02	276.25 ± 58.75	-	-	<0.001	-	-	-
Dave et al. [25]	219.25 ± 42.90	-	-	-	-	230.67 ± 44.50 (MCT)	279 ± 37.52	Also investigated non-inflammatory active TAO and GO without TAO
Kurt et al. [29]	270.47 ± 60.35	305.53 ± 87.93	-	-	0.013	-	--	TAO group was composed of inactive patients
Lai et al. [30]	-	-	287.50 ± 78.55	331.29 ± 83.67	0.001	-	-	-
Noce et al. [34]	-	-	135.89 ± 19.80	285.62 ± 32.5	0.0089	-	-	Also assessed mild/moderate/severe groups of patients
Yu et al. [40]	-	-	256.33 ± 50.18	313.47 ± 100.32	0.003	-	-	-
Zhong et al. [42]	-	-	223.56 ± 78.69	257.33 ± 97.40	0.03	276.23 ± 84.01	224.68 ± 111.61	Active vs. inactive: *p*-value = 0.049; active vs. control *p* value: 0.010; inactive vs. control: *p* value = 1

Abbreviations: GO—Graves’ orbitopathy; SFCT—subfoveal choroidal thickness; and TAO—thyroid-associated orbitopathy.

**Table 6:** Studies investigating GCC/GCL in thyroid-associated ophthalmopathy.

Study	GCL Control Group	GCL TAO Group	GCC Control Group	GCC TAO Group	*p* Value	Observations
Casini et al. [23]	18.30 ± 4.99	14.87 ± 3.0	-	-	0.001	Values for the central part
Guo et al. [27]	87.1 ± 3.8	86.4 ± 5.7 (mild)/82.8 ± 3.8 (moderate to severe)/77.5 ± 10 (DON)	-	-	<0.001 (moderate to severe/DON versus control)	No significant difference between mild and control
Luo et al. [31]	-	-	100.54 ± 8.21	101.77 ± 5.18 (mild)/101.47 ± 7.27 (moderate to severe)/100.10 ± 8.16 (DON)	>0.05	No significant difference between any of the groups
Ogmen et al. [35]	17.5	14	-	-	0.02	GCL thinner in patients with GO than control, but similar in patients without GO and controls
Wu et al. [38]	14.6 ± 4.2	12.0 ± 4.1 (non-DON)/12.0 ± 3.4 (DON)	-	-	0.005	Investigated GCL + IPL
Zhang et al. [41]	100.30 ± 7.43	98.46 ± 14.53 (non-DON)/90.52 ± 11.89 (DON)	-	-	0.008	Compared with non-DON eyes, DON eyes had thinner mean GCC thickness
Zhong et al. [42]	68 ± 6	69 ± 6	-	-	1	Same values for active and inactive

Abbreviations: GCC—ganglion cell complex; GCL—ganglion cell layer; and IPL—inner plexiform layer.

## Data Availability

No new data created. Tables and analyses can be retrieved from the corresponding author.

## Supplementary Materials

The following supporting information can be downloaded at https://www.mdpi.com/article/10.3390/life15020293/s1, Table S1: PRISMA checklist; Table S2: Newcastle-Ottawa risk assessment for cross-sectional studies; Table S3: Newcastle-Ottawa risk assessment for case–control studies; and Table S4: Newcastle-Ottawa risk assessment for cohort studies.
