# Supplementary material for: Retinal and Choroidal Alterations in Thyroid-Associated Ophthalmopathy: A Systematic Review
Source: Life (Basel). 2025 Feb 13;15(2):293. doi: 10.3390/life15020293 (PMC11856781; doi:10.3390/life15020293)
Supplement: Supplementary file 1 [file life-15-00293-s001.zip › life-3449432-supplementary.pdf]

Section and Topic	Item #	Checklist item	Location where item is reported
**TITLE**			
Title	1	Identify the report as a systematic review.	Lines 2-3
**ABSTRACT**			
Abstract	2	See the PRISMA 2020 for Abstracts checklist.	Lines 15-30
**INTRODUCTION**			
Rationale	3	Describe the rationale for the review in the context of existing knowledge.	Lines 77-83
Objectives	4	Provide an explicit statement of the objective(s) or question(s) the review addresses.	Lines 92-97
**METHODS**			
Eligibility criteria	5	Specify the inclusion and exclusion criteria for the review and how studies were grouped for the syntheses.	Table 1
Information sources	6	Specify all databases, registers, websites, organisations, reference lists and other sources searched or consulted to identify studies. Specify the date when each source was last searched or consulted.	Lines 108-109
Search strategy	7	Present the full search strategies for all databases, registers and websites, including any filters and limits used.	Lines 107-115
Selection process	8	Specify the methods used to decide whether a study met the inclusion criteria of the review, including how many reviewers screened each record and each report retrieved, whether they worked independently, and if applicable, details of automation tools used in the process.	Lines 127-130
Data collection process	9	Specify the methods used to collect data from reports, including how many reviewers collected data from each report, whether they worked independently, any processes for obtaining or confirming data from study investigators, and if applicable, details of automation tools used in the process.	Lines 143-154
Data items	10a	List and define all outcomes for which data were sought. Specify whether all results that were compatible with each outcome domain in each study were sought (e.g. for all measures, time points, analyses), and if not, the methods used to decide which results to collect.	Lines 146
	10b	List and define all other variables for which data were sought (e.g. participant and intervention characteristics, funding sources). Describe any assumptions made about any missing or unclear information.	-
Study risk of bias assessment	11	Specify the methods used to assess risk of bias in the included studies, including details of the tool(s) used, how many reviewers assessed each study and whether they worked independently, and if applicable, details of automation tools used in the process.	Lines 156-160
Effect measures	12	Specify for each outcome the effect measure(s) (e.g. risk ratio, mean difference) used in the synthesis or presentation of results.	Lines 167-170
Synthesis methods	13a	Describe the processes used to decide which studies were eligible for each synthesis (e.g. tabulating the study intervention characteristics and comparing against the planned groups for each synthesis (item #5)).	Lines 162-166
	13b	Describe any methods required to prepare the data for presentation or synthesis, such as handling of missing summary statistics, or data conversions.	-
	13c	Describe any methods used to tabulate or visually display results of individual studies and syntheses.	Lines 172-173

Section and Topic	Item #	Checklist item	Location where item is reported
	13d	Describe any methods used to synthesize results and provide a rationale for the choice(s). If meta-analysis was performed, describe the model(s), method(s) to identify the presence and extent of statistical heterogeneity, and software package(s) used.	Lines 162-166
	13e	Describe any methods used to explore possible causes of heterogeneity among study results (e.g. subgroup analysis, meta-regression).	-
	13f	Describe any sensitivity analyses conducted to assess robustness of the synthesized results.	Lines 170-172
Reporting bias assessment	14	Describe any methods used to assess risk of bias due to missing results in a synthesis (arising from reporting biases).	Lines 174-176
Certainty assessment	15	Describe any methods used to assess certainty (or confidence) in the body of evidence for an outcome.	-
**RESULTS**			
Study selection	16a	Describe the results of the search and selection process, from the number of records identified in the search to the number of studies included in the review, ideally using a flow diagram.	Figure 1
	16b	Cite studies that might appear to meet the inclusion criteria, but which were excluded, and explain why they were excluded.	-
Study characteristics	17	Cite each included study and present its characteristics.	Table 3
Risk of bias in studies	18	Present assessments of risk of bias for each included study.	Tables S2-S4
Results of individual studies	19	For all outcomes, present, for each study: (a) summary statistics for each group (where appropriate) and (b) an effect estimate and its precision (e.g. confidence/credible interval), ideally using structured tables or plots.	Tables 3-6
Results of syntheses	20a	For each synthesis, briefly summarise the characteristics and risk of bias among contributing studies.	Tables 4-6
	20b	Present results of all statistical syntheses conducted. If meta-analysis was done, present for each the summary estimate and its precision (e.g. confidence/credible interval) and measures of statistical heterogeneity. If comparing groups, describe the direction of the effect.	-
	20c	Present results of all investigations of possible causes of heterogeneity among study results.	Lines 451-457
	20d	Present results of all sensitivity analyses conducted to assess the robustness of the synthesized results.	Lines 263-267
Reporting biases	21	Present assessments of risk of bias due to missing results (arising from reporting biases) for each synthesis assessed.	-
Certainty of evidence	22	Present assessments of certainty (or confidence) in the body of evidence for each outcome assessed.	-
**DISCUSSION**			
Discussion	23a	Provide a general interpretation of the results in the context of other evidence.	Lines 382-390

Section and Topic	Item #	Checklist item	Location where item is reported
	23b	Discuss any limitations of the evidence included in the review.	Lines 451-462
	23c	Discuss any limitations of the review processes used.	Lines 462-464
	23d	Discuss implications of the results for practice, policy, and future research.	Lines 388-390
**OTHER INFORMATION**			
Registration and protocol	24a	Provide registration information for the review, including register name and registration number, or state that the review was not registered.	Lines 101-102
	24b	Indicate where the review protocol can be accessed, or state that a protocol was not prepared.	Lines 101-102
	24c	Describe and explain any amendments to information provided at registration or in the protocol.	-
Support	25	Describe sources of financial or non-financial support for the review, and the role of the funders or sponsors in the review.	Lines 479-481
Competing interests	26	Declare any competing interests of review authors.	Line 487
Availability of data, code and other materials	27	Report which of the following are publicly available and where they can be found: template data collection forms; data extracted from included studies; data used for all analyses; analytic code; any other materials used in the review.	Lines 485-486

Table S1. PRISMA Checklist

From: Page MJ, McKenzie JE, Bossuyt PM, Boutron I, Hoffmann TC, Mulrow CD, et al. The PRISMA 2020 statement: an updated guideline for reporting systematic reviews. BMJ 2021;372:n71. doi: 10.1136/bmj.n71. This work is licensed under CC BY 4.0. To view a copy of this license, visit https://creativecommons.org/licenses/by/4.0/

Table S2.Newcastle-Ottawa risk assessment for cross-sectional studies

Study	Representativeness of sample	Non-respondents	Ascertainment of exposure	Comparability	Assesement of outcome	Statistical test	Overall
Casini	Low	Low	Low	Low	Low	Low	Low
Caliskan	Low/Moderate	Moderate	Low	Moderate	Low	Low	Moderate
Dave	Low	Low	Low	Moderate	Low	Low	Low
Elsamkary	Low	Low	Low	Moderate	Low	Low	Low
Jian	Low/Moderate	Low	Low	Low	Low	Low	Low
Kurt	Low/Moderate	Low	Low	Moderate	Low	Low	Low/Moderate
Lai	Low	Moderate	Low	Low	Low	Low	Low
Noce	Low	Low	Low	Moderate	Low	Moderate	Low/Moderate

Park	Low	Moderate	Low	Moderate	Low	Low	Low/Moderate
Sayin	Low	Low	Low	Moderate	Low	Low	Low
Yu	Low	Moderate	Low	Moderate	Low	Moderate	Low/Moderate
Zhong	Low	Low	Low	Moderate	Low	Low	Low

Table S3. Newcastle-Ottawa risk assessment for case control studies

Study	Representativeness of cases	Selection of controls	Definition of controls	Comparability of cases and controls	Exposure ascertainment	Blinding of outcome assesement	Non-response rate	Outcome assesement	Adequacy of follow up	Overall
Guo	Low/Moderate	Low	Low	Moderate	Low	Low/Moderate	Low	Low	Low	Low/Moderate
Luo	Low	Moderate	Low	Moderate	Low	Low	Low	Low	N/A	Low/Moderate
Meirovitch	Low	Low	Low	Low	Low	Moderate	Low	Low	Moderate	Low
Mugdha	Low	Low	Low	Low	Low	Moderate	Low	Low	Low	Low
Zhang	Low/Moderate	Low	Low	Moderate	Low	Low	Low	Low	Low	Low

Table S4. Newcastle-Ottawa risk assessment for cohort studies studies

Study	Representativeness	Selection	Ascertnaiment of exposure	Comparability	Assessment of outcome	Follow-up	Adequacy of follow up	Overall
Abdolizadeh	Low	Low	Low	Moderate	Low	Moderate	Moderate	Moderate
Dai	Low	Low	Low	Low	Low	Low	Low	Low
Ogmen	Low	Low	Low	Moderate	Low	Low	Low	Low
Wu	Low	Low	Low	Low	Low	Low	Low	Low
Xu	Low	Low	Low	Moderate	Low	Low	Low	Low
